# Integrative Description and Redescription of Black Fly (Diptera: Simuliidae) Species in the *Simulium* (*Gomphostilbia*) *ceylonicum* Species-Group from Thailand †

**DOI:** 10.3390/insects16101034

**Published:** 2025-10-08

**Authors:** Sorawat Thongsahuan, Kittipat Aupalee, Afham Yakoh, Domechai Kaewnoi, Wanchai Maleewong, Wichai Srisuka, Anchalee Wannasan, Atiporn Saeung, Hiroyuki Takaoka

**Affiliations:** 1 Faculty of Veterinary Science, Prince of Songkla University, Songkhla 90110, Thailand; sorawat.t@psu.ac.th (S.T.); 6311710032@psu.ac.th (A.Y.); domechai.k@psu.ac.th (D.K.); 2 Parasitology and Entomology Research Cluster (PERC), Department of Parasitology, Faculty of Medicine, Chiang Mai University, Chiang Mai 50200, Thailand; kittipat.aupalee@gmail.com (K.A.); anchalee.wa@cmu.ac.th (A.W.); 3 Department of Parasitology, Faculty of Medicine, Khon Kaen University, Khon Kaen 40002, Thailand; wanch_ma@kku.ac.th; 4 Entomology Section, Queen Sirikit Botanic Garden, Chiang Mai 50180, Thailand; wsrisuka@gmail.com; 5 Tropical Infectious Diseases Research and Education Centre (TIDREC), Higher Institution Centre of Excellence (HICoE), Universiti Malaya, Kuala Lumpur 50603, Malaysia; takaoka@oita-u.ac.jp

**Keywords:** biting flies, *COI*, Diptera, insects, morphology, new species, taxonomy

## Abstract

Utilizing the *COI* barcoding approach, cryptic diversity has previously been detected within the morphologically recognized *Simulium* (*Gomphostilbia*) *trangense* Jitklang, Kuvangkadilok, Baimai, Takaoka & Adler, 2008 and *S.* (*G*.) *sheilae* Takaoka & Davies, 1995, of the *S.* (*G.*) *ceylonicum* species-group. Here, an unknown black fly species belonging to the *S. ceylonicum* species-group from southern Thailand was discovered and described as a new species, *S.* (*G.*) *sipoense* sp. nov. In addition, *S.* (*G.*) *trangense* is herein fully redescribed based on specimens collected from its type locality. Based on an integrative taxonomic approach combining morphological and molecular data, the validity of the newly described *S. sipoense* sp. nov. and the redescribed *S. trangense* is confirmed. Comparative morphological characteristics and phylogenetic analysis, inferred from *COI* sequences, suggest that the new species is conspecific with the species redescribed as *S. trangense*, using specimens collected from Malaysia, and is morphologically and phylogenetically closely related to *S. sheilae*, particularly to the specimens from Indonesia. The redescribed *S. trangense* is genetically highly similar or even identical to the species that was apparently misidentified as *S. sheilae* from southern and western Thailand, and is morphologically very similar to the new species, from which it is clearly distinguished by the relative length of the female claw tooth, shape of the male ventral plate, and color of the larval body. A detailed information on the morphological characteristics separates the new species, and the redescribed *S. trangense* from all other known species of the same species-group in Thailand and neighboring countries is provided. Further studies are warranted to clarify the taxonomic status of several cryptic species recognized within the morphologically defined *S. trangense* and *S. sheilae*.

## Simple Summary

The morphological similarity among black flies often leads to misidentification, particularly among closely related species. By using an integrative taxonomic approach, the species status of black flies can be clarified, and sometimes resulting in the discovery of new species. So far, the *COI* DNA barcoding has revealed several cryptic species within two nominal species, *Simulium trangense* and *S. sheilae*, of the *S. ceylonicum* species-group. Through combining detailed morphological examination of all life stage above the egg with DNA analysis, we successfully redescribed *S. trangense*, which was originally described briefly from Thailand and later fully redescribed from Malaysian specimens based solely on morphological characteristics. Furthermore, we fully described a new species, formally named *S. sipoense* sp. nov. from southern Thailand. The new species is conspecific with the species redescribed as *S. trangense* from Malaysia, as supported by both morphological and molecular evidence. This study represents an important case in black fly taxonomy, highlighting that both the redescription of valid species and the description of new species should be undertaken with caution, using an integrated approach that combines morphological and molecular data.

## 1. Introduction

The *Simulium ceylonicum* species-group, redefined by Takaoka [1], is the fourth largest among 16 species-groups of the subgenus *Gomphostilbia*. This species-group comprises 26 named species and two unnamed species, most of which are distributed in the Oriental Region, while five occur in the Palearctic region [2]. In Thailand, five species of the *S. ceylonicum* species-group have been documented, including *S. curtatum* Jitklang, Kuvangkadilok, Baimai, Takaoka & Adler, 2008; *S. pangsidaense* Takaoka, Srisuka & Saeung, 2021; *S. sheilae*, *S. trangense*, and *S. wijiti* Srisuka, Takaoka & Saeung, 2023 [2]. A previous DNA barcoding study revealed high genetic divergence between the two populations (Thailand and Malaysia) of the morphologically defined *S. trangense*, suggesting that they represent two genetically distinct species [3]. Furthermore, *COI* barcoding followed by species delimitation analyses of Indonesian black flies identified up to four cryptic species within the morphospecies *S. sheilae*, which are generally indistinguishable morphologically but are genetically distinct from the type locality in Malaysia [4].

To date, the medical and veterinary significance of species members of the *S. ceylonicum* species-group remains undetermined due to the lack of information on their biting habits and potential roles as vectors in pathogen transmission [5].

During a recent survey of black fly fauna in the southern part of Thailand, one unnamed black fly species, assigned to the *S. ceylonicum* species-group, was discovered. It is herein described as new species, *S. sipoense* sp. nov. Molecular and morphological analyses reveal that the new species is conspecific with the species redescribed as *S. trangense*, based on specimens collected from Langkawi Island, Malaysia [6], but differs from the true *S. trangense* from its type locality in Thailand. Accordingly, the morphological characteristics and *COI* gene sequences of the new species were analyzed and compared with those of related species within the *S. ceylonicum* species-group from Thailand and neighboring countries.

Additionally, *S. trangense* is redescribed based on specimens collected from its type locality, Huai Yang waterfall in Prachuap Khiri Khan province, western Thailand, since the original description was brief [7]. Morphological and molecular comparisons with related species within the *S. ceylonicum* species-group are also provided.

## 2. Materials and Methods

### 2.1. Morphological Analysis

The materials used for morphological analysis consisted of 6 adults (3 females and 3 males) and 7 mature larvae for *S. sipoense* sp. nov. and 7 adults (2 females and 5 males) and 9 mature larvae for *S. trangense*, with all adults reared from pupae with their associated pupal exuviae and cocoons. Since all specimens were preserved in 80% ethanol, their coloration is described based on their appearance in this preservative. The methods of collection and description, as well as the terminology for morphological features used in this study, followed those of Takaoka [8] and, in part, Adler et al. [9]. Specimen preparation for imaging followed the protocol of Takaoka [10], while the method for capturing photographs adhered to Srisuka et al. [11]. The holotype and paratypes of the new species are deposited in the Entomology Section, Queen Sirikit Botanic Garden (QSBG), Chiang Mai, Thailand.

### 2.2. Molecular Analysis

To investigate the genetic relationships of the new species, the redescribed *S. trangense*, and other related species in the *S. ceylonicum* species-group, we employed the *COI* gene-based DNA barcoding. Two specimens of *S. sipoense* sp. nov. [two larvae (NWCL1-L1 and NWCL1-L2)] and three specimens of *S. trangense* [one female (PKTG1-F1), one male (PKTG1-M1), and one larva (PKTG1-L1)] were randomly selected for molecular analysis. DNA was extracted from each individual specimen using either thorax of adult fly or abdominal segments 1–5 of larva, with the PureLink^®^ Genomic DNA Mini Kit (Invitrogen, Waltham, MA, USA). The universal primers, LCO1490 and HCO2198, were used for amplifying a 658 bp fragment of the *COI* gene [12]. *COI* gene amplification followed the PCR protocol described previously by Srisuka et al. [11]. After PCR amplifications, PCR products were checked using 1.5% agarose gel electrophoresis. Samples successfully amplified with an expected DNA fragment of ~700 bp were purified and sequenced at First BASE Laboratories Sdn Bhd (Seri Kembangan, Malaysia) using the same primers as for PCR.

Raw sequences of each black fly specimen were checked for quality and then assembled to obtain a consensus sequence using Geneious Prime 2025.0.4 [13]. All *COI* sequences (accession nos. PV177263–PV177265 for *S. trangense* and PV938055 and PV938056 for *S. sipoense* sp. nov.) generated were compared with those of the *S. ceylonicum* species-group deposited in the NCBI GenBank using the Basic Local Alignment Search Tool (BLAST) [14], available at https://blast.ncbi.nlm.nih.gov/Blast.cgi (accessed on 7 July 2025). Furthermore, genetic differentiation among species in the *S. ceylonicum* species-group was calculated using the Kimura 2-parameter (K2P) model in MEGA 12 program [15,16].

To examine genetic relationships among species in the *S. ceylonicum* species-group, phylogenetic trees of the *COI* gene sequences were constructed based on neighbor-joining (NJ), maximum likelihood (ML), and Bayesian inference (BI) methods. Both NJ and ML trees were built using MEGA 12 software, with branch support based on 1000 bootstrap samples [16,17]. BI was constructed with MrBayes v.3.2.7 [18] and run for two million generations, with sampling every 100 generations and a burn-in of 25%. The best-fit substitution model for ML and BI, which is GTR+G+I, was determined by jModelTest v.2.1.10 [19] based on the Bayesian Information Criterion (BIC). Representative *COI* sequences of six other related species in the *S. ceylonicum* species-group (*S. sheilae*, *S. curtatum*, *S. rangatense* Takaoka, Sofian-Azirun & Wayan, 2017, *S. leparense* Takaoka, Sofian—Azirun & Ya’cob, 2014, *S. wijiti*, and *S. trangense*) available on the GenBank database were fetched and included in the phylogenetic analyses (Table 1).

### 2.3. Ethics Statement

The study protocol was reviewed and approved by the Research Ethics Committee (Institutional Animal Care and Use Committee) of the Faculty of Medicine, Chiang Mai University, Chiang Mai province, Thailand (Protocol No. 46/2561).

## 3. Results

### 3.1. Species Description

*Simulium* (*Gomphostilbia*) *sipoense* Srisuka, Takaoka & Saeung sp. nov.

*Simulium* (*Gomphostilbia*) *trangense* (nec Jitklang, Kuvangkadilok, Baimai, Takaoka & Adler, 2008.): Takaoka et al. 2018: 21–27 (female, male, pupa, and mature larva).

#### 3.1.1. Diagnosis

**Figure 1:**
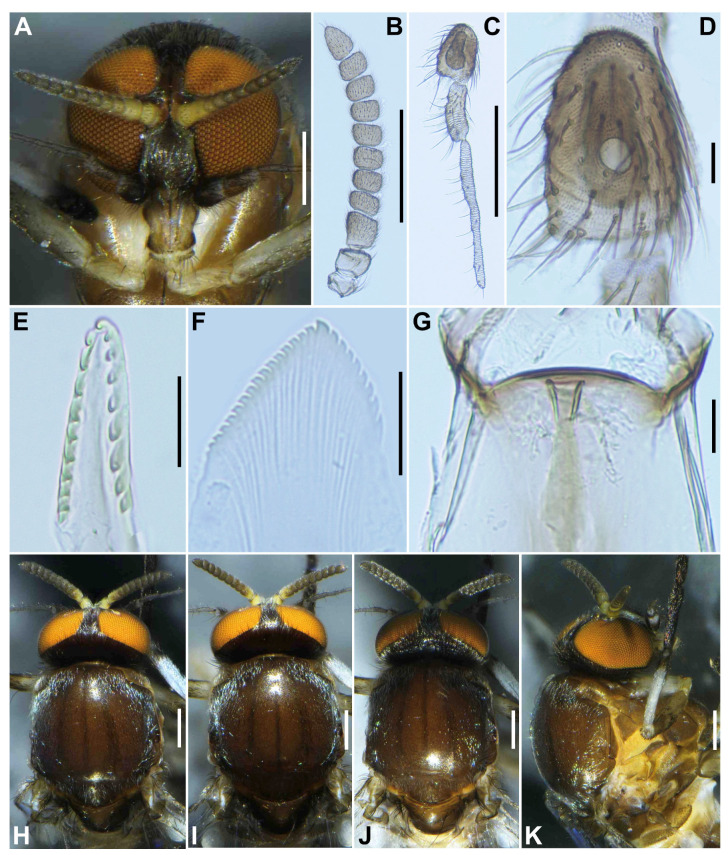
Female of *S*. *sipoense* sp. nov. (**A**) Head, front view; (**B**) Antenna; (**C**) Maxillary palpus, segments 3–5; (**D**) Third palpal segment with sensory vesicle; (**E**) Lacinia; (**F**) Mandible; (**G**) Cibarium; (**H**–**K**) Scuta, dorsal (**H**–**J**), and lateral (**K**) view, illuminated dorsomedially (**H**), anteriorly (**I**), and posteriorly (**J**). Scale bars: 0.2 mm for (**A**–**C**,**H**–**K**); and 0.02 mm for (**D**–**G**).

**Figure 2:**
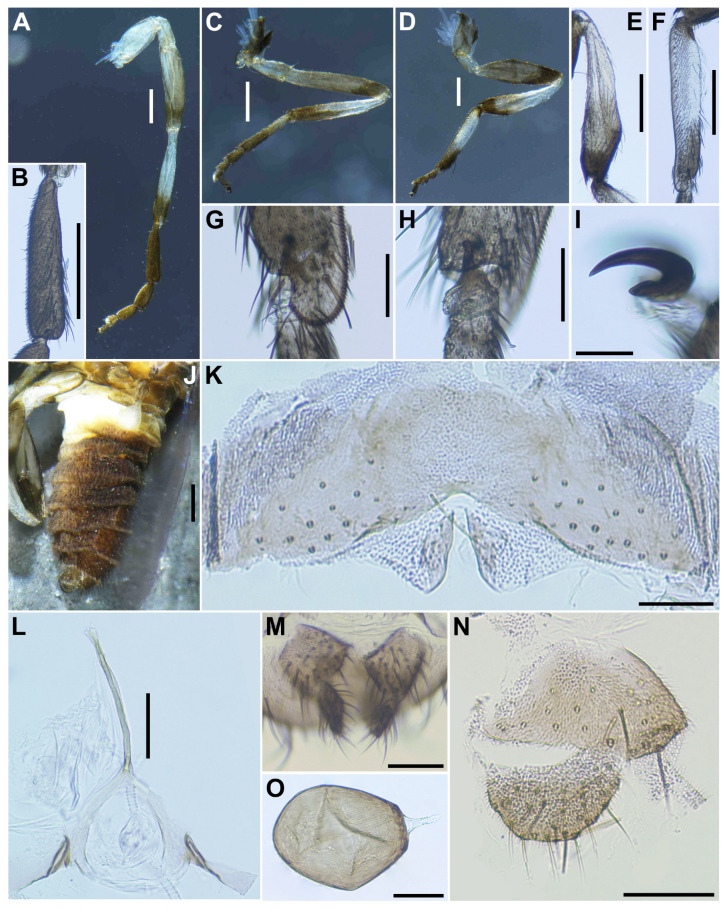
Female of *S*. *sipoense* sp. nov. (**A**) Foreleg; (**B**) Fore basitarsus; (**C**) Midleg; (**D**) Hind leg; (**E**) Hind tibia, left side, outer view; (**F**) Hind basitarsus and second tarsomere, left side, outer view; (**G**) Calcipala, left side, inner view; (**H**) Pedisulcus, left side, outer view; (**I**) Claw; (**J**) Abdomen, lateral view; (**K**) Sternite 8 and ovipositor valves, ventral view; (**L**) Genital fork, ventral view; (**M**,**N**) Paraproct and cercus, ventral (**M**) and lateral (**N**) view; (**O**) Spermatheca. Scale bars: 0.2 mm for (**A**–**F**,**J**); 0.05 mm for (**G**,**H**,**K**–**O**); and 0.02 mm for (**I**).

The new species is distinguished from all other known species in the *S. ceylonicum* species-group based on the combination of the following morphological characteristics. Female: Long sensory vesicle, 0.65 time as long as third palpal segment (Figure 1D), claw with short basal tooth, 0.45 times as long as claw (Figure 2I). Male: Upper-eye (large) facets in 10 vertical columns and 12 horizontal rows; hind basitarsus enlarged, 3.1 times as long as its greatest width (Figure 4F), ventral plate parallel-sided and with its posterior margin slightly convex medially when viewed ventrally (Figure 4K). Pupa: Gill with 8 long thread-like filaments arranged as 3+3+2 from dorsal to ventral (Figure 6A); abdomen transparent, and with pair of wide, plate-like terminal hooks with their outer margins crenulated (Figure 6J); cocoon slipper-shaped, without an anterodorsal projection. Mature larva: Thoracic segment 1 with broad gray transverse band and abdominal segments 2–8 each with reddish-brown markings (Figure 7A,B), postgenal cleft deep (Figure 8G), but not reaching posterior margin of hypostoma.

#### 3.1.2. Morphological Description

**Female** (*n* = 3). Body length 2.1–2.3 mm (mean 2.2 mm). ***Head*** (Figure 1A). Slightly narrower than width of thorax. Frons brownish black to black, moderately covered with yellowish white scale-like recumbent short hairs interspersed with several dark longer hairs along each lateral margin; frontal ratio 1.3–1.4:1.0:2.1–2.2; frons-head ratio 1.0:4.6–4.7. Fronto-ocular area well developed, narrow, directed dorsolaterally. Clypeus brownish black to black, shiny, silver pruinose, brilliantly iridescent when illuminated, densely covered with yellowish white short hairs interspersed with dark longer hair on each side. Labrum 0.6–0.7 times as long as clypeus. Antenna (Figure 1A,B) composed of scape, pedicel, and 9 flagellomeres, brownish black except scape, pedicel, and anterior surface of first and second flagellomere yellow, first flagellomere 1.7–1.8 times as long as second. Maxillary palpus composed of 5 segments, medium brown, proportional lengths of third, fourth, and fifth segments 1.0:1.0–1.1:2.7–2.8; third segment (Figure 1C) swollen; sensory vesicle (Figure 1D) elongate, 0.65 times as long as third segment, with medium-sized opening. Maxillary lacinia (Figure 1E) with 8 inner and 11 outer teeth. Mandible (Figure 1F) with 17 or 18 inner and 8 or 9 outer teeth. Cibarium (Figure 1G) medially forming sclerotized plate folded forward from posterior margin, with dark mediolongitudinal ridge having bifid apex. ***Thorax*.** Scutum (Figure 1H–K) brownish black to black except anterolateral calli light brown, whitish-pruinose and shiny when illuminated at certain angles, with 3 longitudinal vitae (1 narrow median and 2 broad submedian), densely covered with whitish scale-like recumbent short hairs sparsely intermixed with brown similar short hairs mainly on longitudinal vittae (Figure 1I). Scutellum (Figure 1J) dark brown, shiny when illuminated at certain angles, covered with dark brown long hairs. Postnotum (Figure 1J) dark brown, gray-pruinose, shiny when illuminated at certain angles, and bare. Pleural membrane (Figure 1K) whitish yellow and bare. Katepisternum dark brown, longer than deep, shiny when illuminated at certain angles, and moderately covered with dark brown hairs.

***Legs***. Foreleg (Figure 2A): coxa whitish yellow; trochanter light brown; femur light brown with apex medium brown (though extreme tip yellow); tibia white with distal 1/3 brownish black, outer surface largely white iridescent when illuminated; tarsus brownish black; basitarsus (Figure 2B) moderately dilated, 4.5 times as long as its greatest width. Midleg (Figure 2C): coxa medium brown except posterolateral surface dark brown; trochanter light brown; femur light to medium brown with apex medium to dark brown (though extreme tip yellow); tibia white on little more than basal half, and light to dark brown on rest; tarsus brownish black, except basal half of basitarsus light brown, though its border not well defined. Hind leg (Figure 2D): coxa light brown; trochanter whitish yellow; femur medium brown with base whitish yellow and apical cap dark brown; tibia (Figure 2E) whitish on basal half or little more, and light brown to brownish black on apical covered with whitish-yellow fine hairs on outer and posterior surfaces of basal three-fourths and white sheen on posterior surface of basal three-fourths when illuminated at certain angles; tarsus brownish black except basal two-thirds of basitarsus (though base light brown and narrow portion along anterior margin slightly darkened), and basal one-third of second tarsomere white; basitarsus (Figure 2F) narrow, nearly parallel-sided, 5.4 times as long as wide, and 0.6 and 0.5 times as wide as greatest widths of tibia and femur, respectively; calcipala (Figure 2G) nearly as long as wide at base, and 0.42 times as wide as greatest width of basitarsus. Pedisulcus (Figure 2H) well defined. Claw (Figure 2I) with large basal tooth 0.45 times length of claw. ***Wing***. (*n* = 4). Length 1.9–2.1 mm (mean 2.0 mm). Costa with dark spinules and dark hairs. Subcosta with dark hairs except near apex bare. Base of radial vein with tuft of dark brown hairs. Basal portion of radius fully haired; R_1_ with dark spinules and hairs; R_2_ with hairs only. Basal cell absent. ***Halter***. White except basal stem darkened. ***Abdomen*** (Figure 2J). Basal scale light brown, with fringe of whitish-yellow hairs. Dorsal surface of abdomen brownish black except segment 2 whitish brown, moderately covered with dark short to long hairs and yellow fine short hairs; tergites of segments 6–9 shiny when illuminated at certain angles; ventral surface of segment 2 whitish, and those of other segments medium to dark brown. Sternal plate on segment 7 undeveloped. ***Terminalia***. Sternite 8 (Figure 2K) bare medially, with 12–17 medium-long to long hairs, together with 2 or 3 slender short hairs on each side. Ovipositor valves (Figure 2K) triangular, thin, membranous, each moderately covered with micro-setae interspersed with 1 or 2 short hairs; inner margins slightly sinuous, sclerotized, and somewhat separated from each other. Genital fork (Figure 2L) of usual inverted-Y form, with slender and thick stem; arms of moderate width and moderately folded medially, and lateral plate of each arm with thin lobe projection directed posteromedially. Paraproct in ventral view (Figure 2M) somewhat widened posteromedially, with 5 or 6 sensilla on anteromedial surface; paraproct in lateral view (Figure 2N) slightly produced ventrally, 0.42–0.44 times as long as wide, with 18–20 medium-long to long hairs on ventral and lateral surfaces. Cercus in lateral view (Figure 2N) short, rounded posteriorly, 0.54–0.56 times as long as wide. Spermatheca (Figure 2O) ellipsoidal, 1.5 times as long as its greatest width, dark brown except duct unpigmented, and with many fissures on surface; internal setae absent.

**Figure 3:**
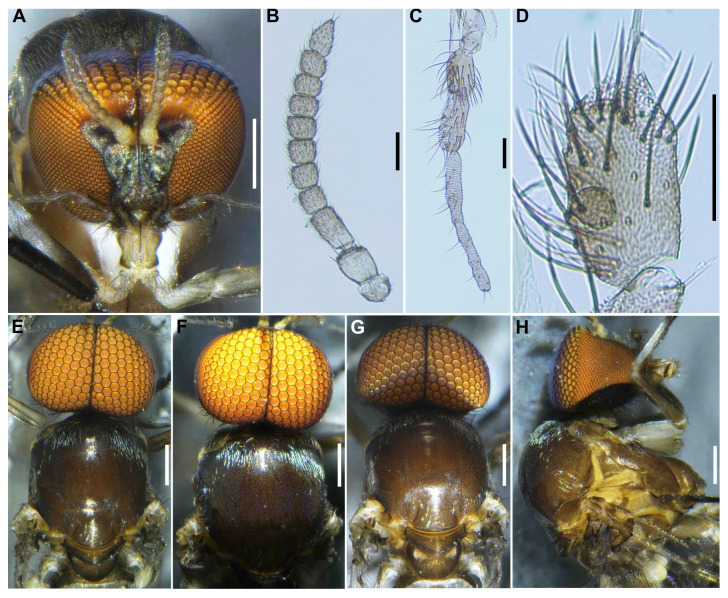
Male of *S*. *sipoense* sp. nov. (**A**) Head, front view; (**B**) antenna; (**C**) Maxillary palpus; (**D**) Third palpal segment with sensory vesicle; (**E**–**H**) Scuta, dorsal (**E**–**G**), and lateral (**H**) view, illuminated dorsomedially (**E**), anteriorly (**F**), and posteriorly (**G**). Scale bars: 0.2 mm for (**A**,**E**–**H**); and 0.05 mm for (**B**–**D**).

**Male** (*n* = 3). Body length 2.6–2.7 mm (mean 2.65 mm). ***Head*** (Figure 3A). Wider than thorax. Upper eye medium brown, consisting of large facets in 10 vertical columns and 12 horizontal rows. Face brownish black, white-pruinose. Clypeus brownish black, whitish-pruinose, densely covered with golden-yellow scale-like medium-long hairs (mostly directed upward) interspersed with several light-brown simple longer hairs. Antenna (Figure 3A,B) composed of scape, pedicel, and 9 flagellomeres, medium to dark brown except scape, pedicel, and base of first flagellomere yellow; first flagellomere elongate, 1.7–1.8 times length of second (Figure 3B). Maxillary palpus (Figure 3C) light to medium brown, with 5 segments, proportional lengths of third, fourth, and fifth segments 1.0:1.1:3.0–3.1; third segment slightly widened apically; sensory vesicle (Figure 3D) globular, small, 0.17 times length of third segment, and with small opening. ***Thorax***. Scutum (Figure 3E–H) brownish black, except shoulders light brown, scutum white pruinose on each shoulder, along lateral margins and on prescutellar area, when illuminated at various angles, scutum densely covered with whitish-yellow recumbent short hairs (Figure 3E–G). Scutellum dark brown with whitish-pruinose on posterior margin, with dark upright hairs and whitish short hairs (Figure 3E,G). Postnotum (Figure 3G), brownish black, with whitish-pruinose, shiny bare. Pleural membrane light brown, shiny when illuminated at certain angles and bare. Katepisternum (Figure 3H) dark brown, shiny when illuminated at certain angles, and moderately covered with fine whitish-yellow hairs.

**Figure 4:**
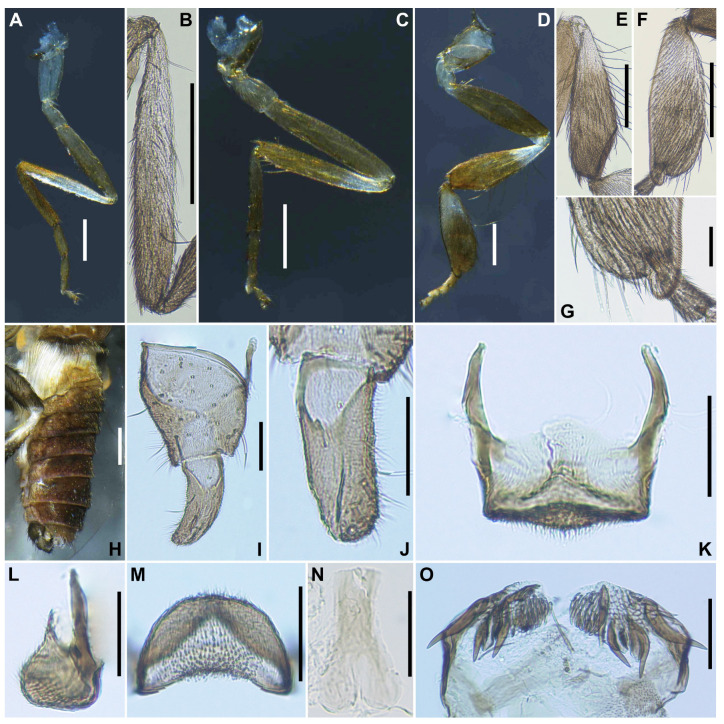
Male of *S*. *sipoense* sp. nov. (**A**) Foreleg; (**B**) Fore tibia; (**C**) Midleg; (**D**) Hind leg; (**E**) Hind tibia; (**F**) Hind basitarsus; (**G**) Calcipala; (**H**) Abdomen, lateral view; (**I**) Coxite and style, ventral view; (**J**) Style, dorsolateral view; (**K**–**M**) Ventral plates, ventral (**K**), lateral (**L**), and caudal (**M**) view; (**N**) Median sclerite; (**O**) Parameres. Scale bars: 0.2 mm for (**A**–**F**,**H**) and 0.05 mm for (**G**,**I**–**O**).

***Legs***. Foreleg (Figure 4A), coxa whitish-yellow; trochanter light brown; femur light brown with apex medium brown; tibia (Figure 4B) light brown on extreme base, dark brown on the rest, outer surface and center area largely white, iridescent when illuminated; tarsus brownish black; basitarsus nearly parallel-sided, 6.0 times as long as its greatest width. Midleg (Figure 4C), coxa medium brown except posterolateral surface dark brown; trochanter light brown; femur light brown with apex medium to dark brown (though extreme tip yellow); tibia whitish-yellow on basal one-fifth, and medium to dark brown on rest; tarsus dark brown to brownish black except basal one-third of basitarsus light to medium brown though its border not well defined. Hind leg (Figure 4D), coxa light to medium brown; trochanter whitish brown; femur medium brown with basal extreme whitish-yellow and apical cap dark brown; tibia (Figure 4E) dark brown to brownish black except basal one-third whitish; tarsus medium to dark brown except basal one-third or little more of basitarsus white (though base medium brown), and basal one-third of second tarsomere white; basitarsus (Figure 4F) enlarged, wedge-shaped, 3.1 times as long as wide, and 0.94 and 1.07 times as wide as greatest width of tibia and femur, respectively; calcipala (Figure 4G) as long as basal width, and 0.27 times as wide as greatest width of basitarsus. Pedisulcus well defined. ***Wing*** (*n* = 4). Length 1.6–1.7 mm (mean 1.65 mm); other characteristics as in female, except subcosta without hairs. ***Halter***. White except basal stem darkened. ***Abdomen*** (Figure 4H). Basal scale dark brown, with fringe of dark brown long hairs. Dorsal surface of abdomen medium brown to brownish black, except segment 2 light brown, moderately covered with dark short to long hairs and yellow short hairs; segments 2 and 5–8 each with pair of shiny dorsolateral patches. ***Genitalia***. Coxite in ventral view (Figure 4I) nearly rectangular, 1.4 times as long as its greatest width. Style in ventral view (Figure 4I) gently bent inward, nearly parallel-sided and with apical spine; style in ventrolateral view (Figure 4J) 2.4 times as long as its greatest width at base, 0.85 times length of coxite, and nearly parallel-sided on basal one-third and gently narrowed toward apex. Ventral plate in ventral view (Figure 4K) with body transverse, 0.5 times as long as wide, with posterior half or little more markedly narrower than basal width, with anterior margin produced anteromedially, and posterior margin slightly convex medially, densely covered with micro-setae on ventral surface; basal arms of moderate length, slightly divergent from base to middle, then somewhat convergent apically; ventral plate in lateral view (Figure 4L) moderately produced ventrally; ventral plate in caudal view (Figure 4M) rounded ventrally, about half as high as basal width, and densely covered with micro-setae on posterior surface. Median sclerite (Figure 4N) wide, plate-like, thin, and well sclerotized on base to middle area, with widened apex. Parameres (Figure 4O) each with 4 long and several short ones. Aedeagal membrane moderately covered with micro-setae. Cercus small, rounded, and encircled with 12–13 hairs.

**Figure 5:**
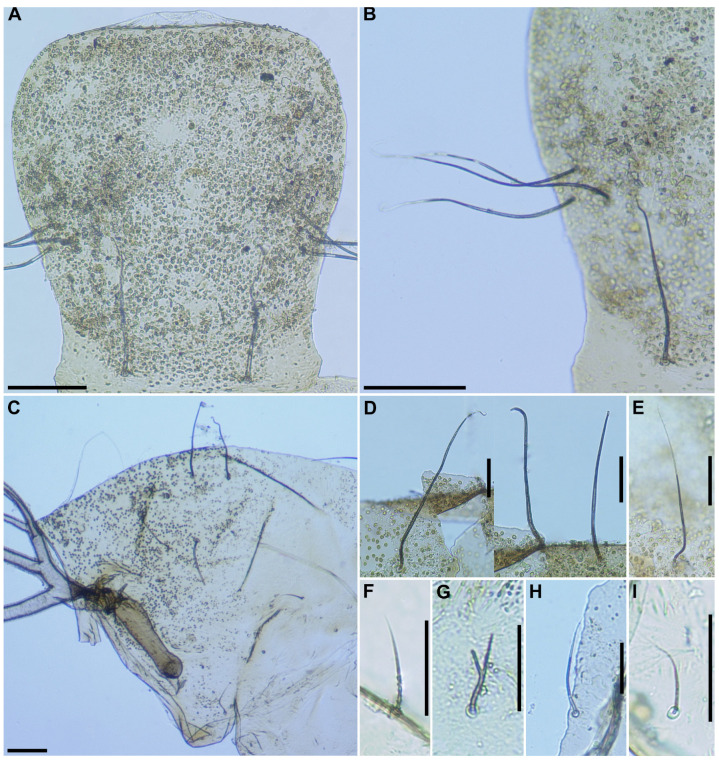
Pupa of *S*. *sipoense* sp. nov. (**A**) Frons and tubercles on frons; (**B**) Frontal trichomes and facial trichome; (**C**) Tubercles on thoracic integument; (**D**–**I**) Thoracic trichomes on anterodorsal (**D**), anterolateral (**E**), mediolateral (**F**,**G**), and ventrolateral (**H**,**I**) surfaces. Two types of trichomes are present on mediolateral surface, including unbranched (**F**) and branched (**G**) trichomes. Scale bars: 0.1 mm for (**A**–**C**) and 0.05 mm for (**D**–**I**).

**Pupa** (*n* = 6). Body length 3.1–3.4 mm (mean 3.3 mm). ***Head***. Integument yellow (Figure 5A), densely covered with small round tubercles, except antennal sheaths. Frons with 3 pairs of unbranched long trichomes (Figure 5B); face with pair of unbranched long trichomes (Figure 5B); 3 frontal trichomes on each side arising close together, subequal in length to one another, and somewhat as long as facial one. ***Thorax***. Integument yellow (Figure 5C), moderately covered by round tubercles on anterior lateral portion and posterior half somewhat sparsely covered with small tubercles, with 3 long dorsomedial trichomes (Figure 5D), 2 long anterolateral trichomes (Figure 5E), 1 unbranched (Figure 5F) or branched (Figure 5G) medium-long mediolateral trichome, and 3 ventrolateral trichomes with uncoiled apices (1 medium-long (Figure 5H), 2 short (Figure 5I)) on each side; all trichomes unbranched.

**Figure 6:**
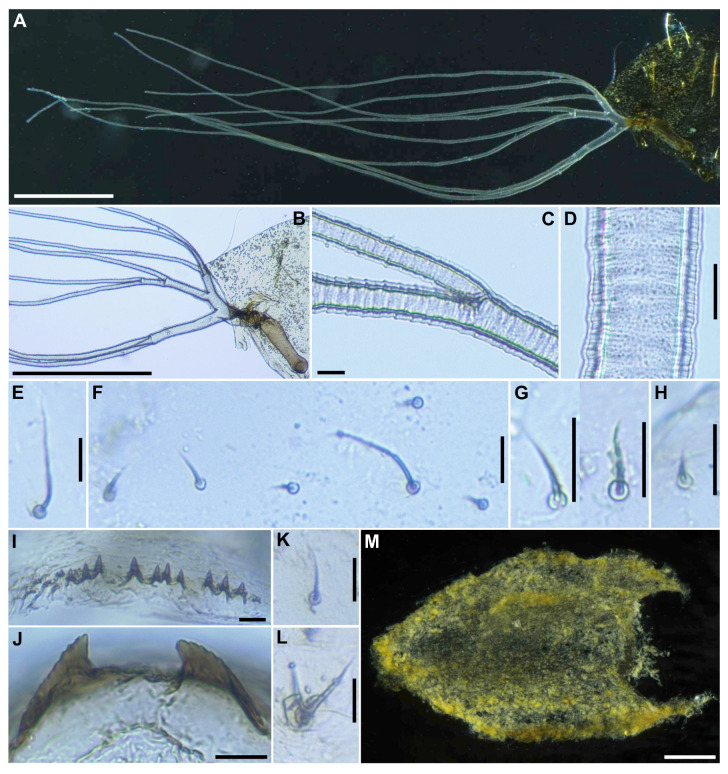
Pupa of *S*. *sipoense* sp. nov. (**A**) Gill filaments; (**B**) Basal portion of gill; (**C**,**D**) Surface of gill filaments; (**E**) Hair-like seta on abdominal segment 1; (**F**) Hair-like seta and 5 short spinous setae on dorsum of second abdominal segment; (**G**,**H**) Setae on dorsum of abdominal segments 3, 4 (**G**), and 5 (**H**); (**I**) Spine-combs on dorsum of abdominal segments 6–9; (**J**) Terminal hooks, caudal view; (**K**) Short seta on ventral surface of abdominal segment 4; (**L**) Bifid hook on ventral surface of abdominal segment 5; (**M**) Cocoon, dorsal view. Scale bars: 0.5 mm for (**A**,**B**,**M**) and 0.02 mm for (**C–L**).

Gill (Figure 6A) composed of 8 slender thread-like filaments, arranged as [(2+1)+(1+2)]+2 from dorsal to ventral, with short common basal stalk (Figure 6B) having somewhat swollen transparent basal fenestra at base; common basal stalk 0.67 times length of interspiracular trunk (Figure 6B); dorsal and middle triplets sharing short stalk; dorsal triplet (Figure 6B) composed of 2 paired and 1 individual filaments with short primary and secondary stalks; middle triplet (Figure 6B) composed of 1 individual and 2 paired filaments with medium-long primary and short secondary stalks; ventral paired filaments (Figure 6B) with long stalk 1.4–1.6 times length of common basal stalk and as long as interspiracular trunk; all filaments light yellow, gradually tapered toward apex; 2 filaments of ventral pair subequal in length (ca. 3.2 mm including their own stalk) to each other, 6 filaments of dorsal and middle triplets subequal in length to one another, and slightly shorter than 2 filaments of ventral pair; filaments of ventral pair subequal in thickness to each other; primary stalk of dorsal triplet lying against stalk of ventral pair at angle of about 80 degrees when viewed laterally; cuticle of all filaments with well-defined annular ridges (Figure 6C), densely covered with micro-tubercles (Figure 6D). ***Abdomen***. Dorsally, all segments unpigmented or light yellow, without micro-tubercles; first segment with 1 slender medium long hair-like seta (Figure 6E) on each side; segment 2 with 1 slender long hair-like seta and 5 short spinous setae submedially on each side (Figure 6F), all setae unbranched; segments 3 and 4 each with 4 hooked spines and 1 unbranched short seta (Figure 6G) on each side; segment 5 with 3 short setae (Figure 6H) submedially on each side; segments 6–9 each with spine-combs in transverse row (Figure 6I) (though those on segment 9 somewhat smaller than those on all other segments) and comb-like groups of micro-spines on each side; segment 9 with pair of wide, plate-like terminal hooks (Figure 6J), with their outer margins 3.3–3.5 times as long as their inner margins and crenulated. Ventrally, segment 4 with 1 unbranched slender short seta (Figure 6K) on each side; segment 5 with pair of bifid hooks (Figure 6L) submedially and few unbranched slender short setae on each side; segments 6 and 7 each with pair of bifid inner and unbranched outer hooks, and few unbranched slender short setae on each side; segments 4–8 with comb-like groups of micro-spines. Each side of segment 9 with 3 grapnel-shaped hooklets. ***Cocoon*** (*n* = 6). Length 2.8–3.1 mm (mean 3.0 mm), width 1.9–2.2 mm (mean 2.6 mm) (Figure 6M). Wall pocket-shaped, moderately woven, somewhat extended ventrolaterally and without anterodorsal portion; posterior half with floor roughly or moderately woven; individual threads visible or not.

**Figure 7:**
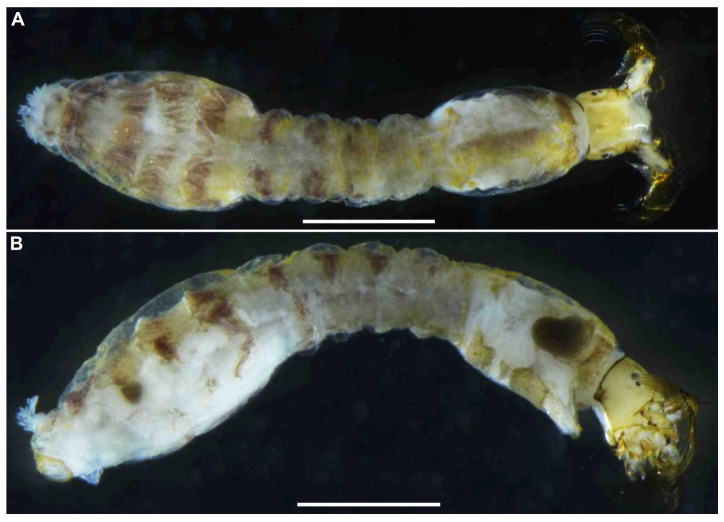
Larva of *S*. *sipoense* sp. nov. (**A**,**B**) Whole body, dorsal (**A**) and lateral (**B**) view. Scale bars: 1 mm.

**Mature larva** (*n* = 7). Body length 4.3–4.8 mm. (mean 4.5 mm) (Figure 7A,B). Body gray except dorsal surface of thorax and part of proleg whitish-gray, ventral surface of thoracic segments 2 and 3 grayish, first thoracic segment with broad brown transverse band unconnected ventrally. Abdominal segments 1 with small reddish-brown spot on each dorsolateral portion, abdominal segments 2–4 each with pair of reddish-brown dorsolateral markings, and abdominal segment 4 also with faint reddish-brown marking ventrally, segments 5–8 each with transverse reddish-brown band on dorsal and dorsolateral surfaces, although those bands on abdominal segments 6–8 often disconnected dorsomedially.

**Figure 8:**
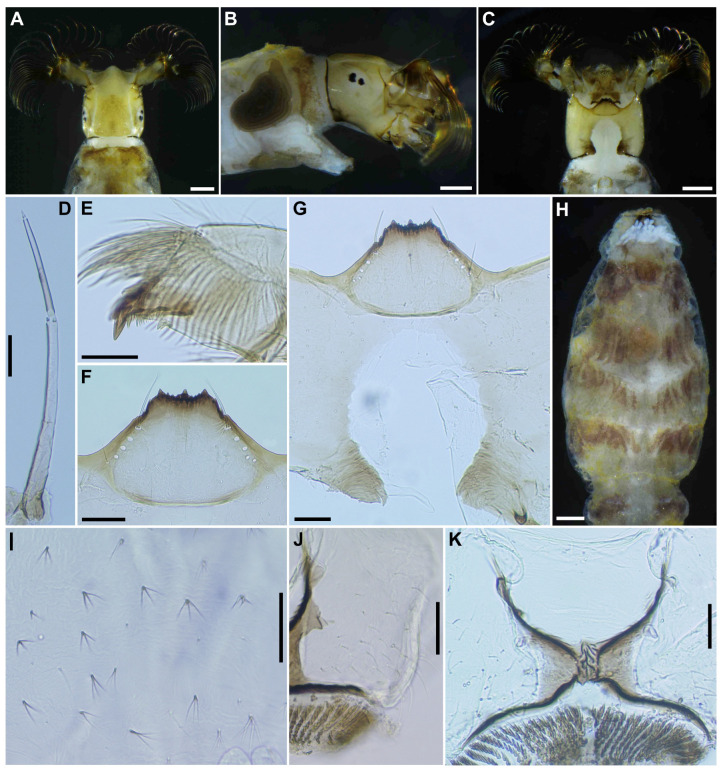
Larva of *S*. *sipoense* sp. nov. (**A**–**C**) Head capsules, dorsal (**A**), lateral (**B**), and ventral (**C**) view; (**D**) Antenna; (**E**) Mandible; (**F**) Hypostoma; (**G**) Postgenal cleft; (**H**,**I**) Setae on abdominal segments 5–8, dorsal view; (**J**) Setae on last abdominal segment; (**K**) Anal sclerite, dorsal view. Scale bars: 0.2 mm for (**A**–**C**,**H**) and 0.05 mm for (**D**–**G**,**I**–**K**).

***Head***. Head capsule width 0.42–0.47 mm. (mean 0.45 mm), length 0.50–0.56 mm. (mean 0.52 mm). Cephalic apotome (Figure 8A) whitish-yellow, head spots faintly positive, sparsely covered with colorless fine setae. Lateral surface (Figure 8B) of head capsule yellow, except posterior portion light brown, eye-spot region whitish, sparsely covered with colorless fine setae. Ventral surface of head capsule (Figure 8C) light yellow, except basal portions of both sides of postgenal cleft dark brown and sparsely covered with minute colorless setae. Antenna (Figure 8D) unpigmented, except first article mostly light brown, composed of 3 articles and apical sensillum, little longer than stem of labral fan; proportional lengths of first, second, and third articles 1.00:1.04–1.06:1.08–1.10. Labral fan with 34–36 primary rays. Mandible (Figure 8E) with 3 comb-teeth decreasing in length from first tooth to third; mandibular serration composed of 1 medium-size and 1 small teeth, major tooth at acute angle against mandible on apical side; supernumerary serration absent. Hypostoma (Figure 8F) with row of 9 apical teeth, of which median tooth nearly as long as or slightly longer than each corner tooth; lateral margin smooth; 5 hypostomal bristles per side lying nearly parallel to lateral margin. Postgenal cleft (Figure 8G) deep, 13–15 times as long as postgenal bridge. Cervical sclerites composed of pair of small light brown rod-like pieces. ***Thorax*** and ***Abdomen***. Thoracic and abdominal cuticle almost bare, except abdominal segments 5–8 (Figure 8H) moderately covered with dark setae, each with 2–4 branches (Figure 8I) and unbranched colorless fine setae dorsolaterally; last abdominal segment moderately covered with long unbranched colorless minute setae (Figure 8J) on dorsolateral surface of each side of anal sclerite. Rectal papilla compound, each of 3 lobes with 6–8 finger-like secondary lobules per lobe. Anal sclerite (Figure 8K) of usual X-form, with anterior arms 0.8–1.0 times as long as posterior ones, broadly sclerotized at base; no sensilla on broad base and posterior to posterior arms; accessory sclerite absent. Last abdominal segment with pair of large conical ventral papillae. Posterior circlet with 69–72 rows of hooklets, with up to 12–13 hooklets per row.

#### 3.1.3. Type Specimens

Holotype. Female (with its associated pupal exuviae and cocoon) (in 80% ethanol), reared from a pupa collected from Sipo waterfall, Ra-Ngae district, Narathiwat province, southern Thailand, 1-VI-2024, by W. Srisuka and S. Thongsahuan. Paratypes. Two females, three males (with their associated pupal exuviae and cocoons), and seven mature larvae (all in 80% ethanol), same data as for the holotype.

#### 3.1.4. Biology

The pupae and larvae of *S. sipoense* sp. nov. were collected from fallen leaves in a streamlet (width 20 cm, depth 5 cm, bed sandy, 21.5˚C, pH 7.3, partially shaded, elevation 210 m, N6°16′7″ E101°38′3″) at the side of a river (width 35 m, depth 40 cm and flow fast). The associated species were *S*. *aureohirtum* Brunetti, 1911 and *S*. *tani* Takaoka & Davies, 1995, complex.

#### 3.1.5. Etymology

The species name *sipoense* refers to Sipo waterfall, the location where this new species was collected.

#### 3.1.6. Remarks

This new species belongs to the *S. ceylonicum* species-group of the subgenus *Gomphostilbia* in the genus *Simulium*, redefined by Takaoka [1]. It is characterized by the dark hair tuft at the base of the radial vein and yellow fore coxae in both female and male. Male also possesses enlarged hind basitarsi (Figure 4F) and ventral plate, with lateral margins nearly parallel-sided or slightly rounded (Figure 4K) when viewed ventrally. The postgenal cleft of the larva is deep, with its apex close to the posterior margin of the hypostoma (Figure 8G).

*Simulium sipoense* sp. nov. is conspecific with the species redescribed as *S. trangense*, based on specimens collected from Langkawi Island, Malaysia [6]. This conclusion is supported by both morphological and genetic similarities. However, Thai specimens (type specimens) exhibit slight morphological differences from the Malaysian specimens (shown in parentheses), particularly in the following numerical features: the length ratio of the female sensory vesicle to the third palpal segment is 0.65 time (0.55–0.59 time), and the ratio of the length of the male hind basitarsus to its greatest width is 3.1 time (3.5–3.9 time).

*Simulium sipoense* sp. nov. is closely similar to *S. sheilae* in sharing the following characteristics: in the female, large sensory vesicle, scutum pattern and claw with large basal tooth; in the male, similar number of upper-eye large facets, small sensory vesicle and ventral plate with its posterior margin slightly convex medially; and in the pupa, similar 8 gill filament arranged as 3+3+2 from dorsal to ventral. However, it is distinguished from *S. sheilae* in the female by the number of teeth on maxillary lacinia, 8 inner and 11 outer teeth (cf. 10 and 13 in *S. sheilae*), and length ratio of the hind basitarsus with its widest 5.4 time (6.3 time in *S. sheilae*); in the male, by the length ratio of the hind basitarsus against its greatest width 3.1 time (3.5 time); in the pupa, by the wide, plate-like terminal hook (cone shaped in *S. sheilae*), cocoon without an anterodorsal projection or bulge when view dorsally (with an anterodorsal projection or bulge in *S. sheilae*); and in the larva, by the number of primary fan rays 34–36 primary rays (cf. 47 primary rays in *S. sheilae*) and first thoracic segment, with dark gray broad transverse band (cf. with a reddish brown broad band) [21].

The new species is distinguished from the three other Thai members (*S*. *curtatum*, *S*. *pangsidaense*, and *S*. *wijiti*) of the *S. ceylonicum* species-group by the following characteristics: in the female, by the length ratio of the sensory vesicle against the third palpal segment 0.65 time (0.60 in *S*. *curtatum*, 0.57–0.63 in *S*. *pangsidaense*, and 0.25–0.32 in *S*. *wijiti*) and the length ratio of the hind basitarsus against its greatest width 5.4 times (5.9 in *S*. *pangsidaense*, 6.3–6.4 in *S*. *wijiti*, and data unavailable for *S*. *curtatum*); in the male, by the upper-eye (large) facets arranged in 10 vertical columns and 12 horizontal rows, (13 columns and 14–16 rows in *S*. *curtatum* and *S*. *pangsidaense*; and 15 columns and 15 or 16 rows in *S*. *wijiti*), hind tibia dark brown to brownish black except the basal one-third whitish (light to medium brown, except the base whitish yellow and the apical cap brownish black in *S. pangsidaense* and yellowish on little more than the basal one-third with an ochreous subbasal marking, and light to medium brown on the rest, except the apical cap brownish black in *S. wijiti* and data unavailable for *S*. *curtatum*), the length ratio of the hind basitarsus against its greatest width is 3.1, compared to 3.8–4.6 in *S*. *pangsidaense* and *S*. *wijiti* (data unavailable for *S*. *curtatum*); in the pupa, by the terminal hook wide and plate-like with its outer margin crenulated (same in *S. pangsidaense*, triangular terminal hooks with their outer margins smooth in *S. wijiti*, and data unavailable for *S*. *curtatum*), and ninth abdominal segment unpigmented or light yellow (dark yellow in *S. pangsidaense*); and in the larva, by the number of the primary rays of the labral fan, 34–36 compared to 27–32 in *S*. *wijiti* and *S*. *pangsidaense* (data unavailable for *S*. *curtatum*), and the first abdominal segment with a small reddish-brown spot on each dorsolateral surface (a reddish-brown transverse band in *S. pangsidaense* and a greenish transverse band in *S. curtatum* and *S. wijiti*) [5,7,21,22].

The new species is distinguished from *S. doisaketense* Jitklang, Kuvangkadilok, Baimai, Takaoka & Adler, 2008, reported from northern Thailand by Jitklang et al. [7], in the larval stage by the presence of reddish-brown transverse bands on abdominal segments 2–4 (cf. *S. doisaketense* has greenish transverse bands on abdominal segments 1–4), and a deep postgenal cleft that is 13–15 times as long as the postgenal bridge (cf. *S. doisaketense* has a medium-long postgenal cleft (1.5–2.8 times as long as postgenal bridge)).

The new species is similar to *S. leparense* from Malaysia in many characteristics, such as the relative length of the female claw tooth; the shape and color of the male hind basitarsus; and a short common stalk of the pupal gill, with 8 filaments and terminal hooks that are wide, plate-like, and have crenulated outer margins. However, *S. sipoense* sp. nov. can be differentiated from *S. leparense* by the following characteristics (characteristics of *S. leparense* in parentheses): in the female, by the length ratio of the sensory vesicle to the third palpal segment of 0.65 (0.55), the color of short hairs on the scutum, which is whitish (dark brown), and scutum pattern with 3 longitudinal vittae (absent); in the male, by the number of upper-eye large facets arranged in 10 vertical columns and 12 horizontal rows (7 or 8 vertical columns and 11 horizontal rows) and length ratio of the sensory vesicle against third palpal segment 0.17 time (0.14 time); and in the pupa, by the presence of 3 grapnel-shaped hooklets on each side of abdominal segment 9 (absent) [23].

### 3.2. Redescription of Species in the Simulium ceylonicum Species-Group

*Simulium* (*Gomphostilbia*) *trangense* Jitklang, Kuvangkadilok, Baimai, Takaoka & Adler, 2008

*Simulium* (*Gomphostilbia*) *trangense* Jitklang et al. 2008: 19–20 (female, male, pupa, and larva).

This species was shortly described based on females, males, pupae, and larvae collected from Huai Yang waterfall on 14-xii-2006 in Prachuap Khiri Khan province, Thailand [7].

#### 3.2.1. Morphological Redescription

**Figure 9:**
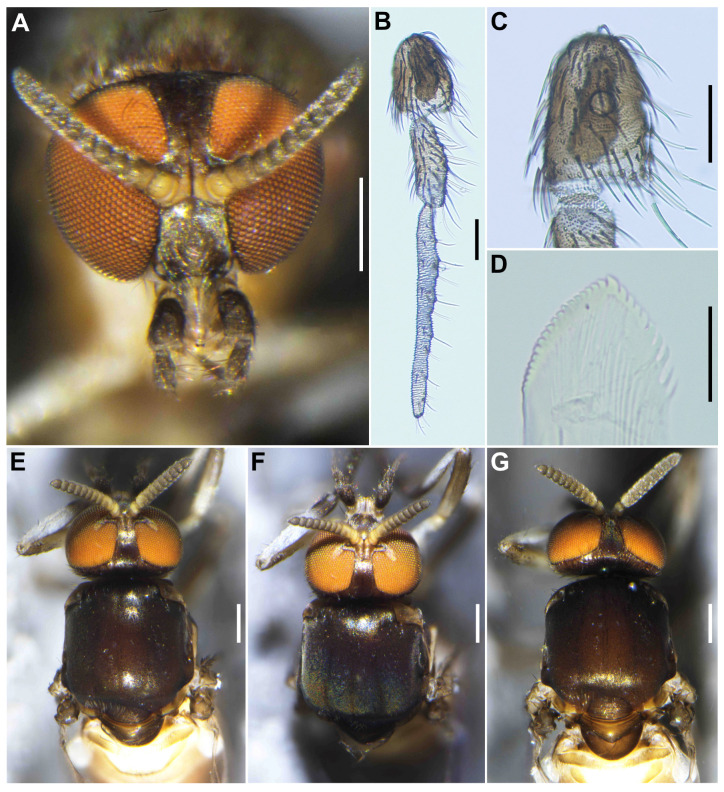
Female of *S*. *trangense.* (**A**) Head, front view; (**B**) Maxillary palpus, segments 3–5; (**C**) Third palpal segment with sensory vesicle, right side, front view. (**D**) Mandible. (**E**–**G**) Scuta, dorsal view, illuminated dorsomedially (**E**), anteriorly (**F**), and posteriorly (**G**). Scale bars: 0.2 mm for (**A**,**E**–**G**); 0.05 mm for (**B**,**C**) and 0.02 mm for (**D**).

**Female** (*n* = 2). Similar to the female of *S*. *sipoense* sp. nov., except in following characteristics. Body length 2.31–2.33 mm (mean 2.3 mm). ***Head*** (Figure 9A). Frontal ratio 1.6–2.0:1.0:1.8–2.1; frons-head ratio 1.0:3.4–3.6. Labrum 0.70–0.79 times as long as clypeus. Antenna composed of scape, pedicel, and 9 flagellomeres, brownish black except scape, pedicel, and anterior surface of basal half of first flagellomere yellow, first flagellomere 2.0–2.1 times as long as second. Maxillary palpus (Figure 9B), proportional lengths of third, fourth, and fifth segments 1.0:1.1:2.6–2.7; third segment (Figure 9C) moderately swollen; sensory vesicle (Figure 9C) elongate, 0.66 times as long as third segment, with medium-sized opening. Maxillary lacinia with 11 inner and 8 or 9 outer teeth. Mandible (Figure 9D) with 18 inner and 9 or 10 outer teeth. ***Thorax*.** Scutum (Figure 9E–G) densely covered with yellowish-white scale-like recumbent short hairs sparsely intermixed with dark similar hairs and without vittae. Scutellum (Figure 9E,F) covered with short white hairs and dark long upright hairs. Postnotum (Figure 9G) brownish black. Pleural membrane white and bare. Katepisternum moderately covered with yellow and dark brown short hairs.

**Figure 10:**
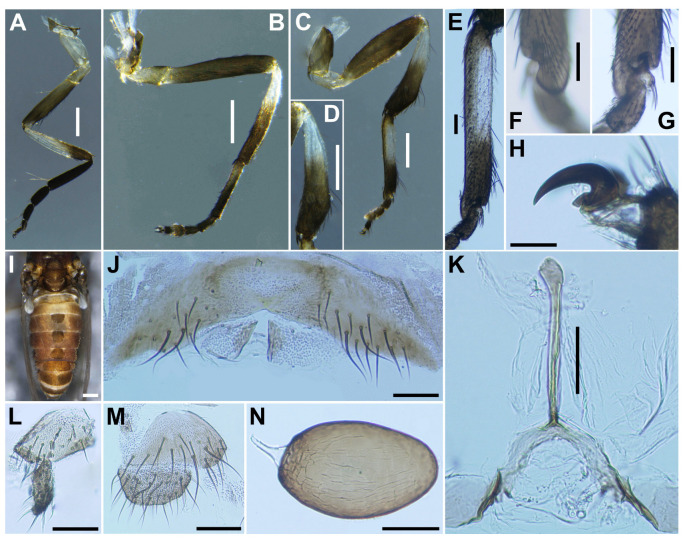
Female of *S. trangense*. (**A**) Foreleg; (**B**) Midleg; (**C**) Hind leg; (**D**) Hind tibia, left side, outer view; (**E**) Hind basitarsus and second tarsomere, left side, outer view; (**F**) Calcipala, left side, inner view; (**G**) Pedisulcus, left side, outer view; (**H**) Claw; (**I**) Abdomen, dorsal view; (**J**) Sternite 8 and ovipositor valves, ventral view; (**K**) Genital fork, ventral view; (**L**,**M**) Paraprocts and cerci, ventral (**L**) and lateral (**M**) view; (**N)** Spermatheca. Scale bars: 0.2 mm for (**A**–**D**,**I**); 0.05 mm for (**E**–**G**,**J**–**N**); and 0.02 mm for (**H**).

***Legs***. Foreleg (Figure 10A): basitarsus moderately dilated, 5.5 times as long as its greatest width. Midleg (Figure 10B): tibia whitish yellow on little more than basal one-third and light to dark brown on the rest. Hind leg (Figure 10C): trochanter light yellow; femur light to medium brown with base whitish yellow and apical cap dark brown; tibia white (Figure 10D) on little less than basal half and light brown to brownish black on apical; tarsus brownish black, except basal two-thirds of basitarsus white (though base light brown and narrow portion along anterior margin slightly darkened), and basal one-third of second tarsomere whitish; basitarsus (Figure 10E) narrow, parallel-sided, 6.8–6.9 times as long as wide, and 0.5–0.6 and 0.5 times as wide as greatest widths of tibia and femur, respectively; calcipala (Figure 10F) longer than wide and 0.55 times as wide as greatest width of basitarsus. Pedisulcus (Figure 10G) well defined. Claw (Figure 10H) with relatively short basal tooth 0.33 times length of claw. ***Wing*** (*n* = 4). Length 2.2–2.6 mm (mean 2.23 mm). ***Abdomen*** (Figure 10I). ***Terminalia***. Sternite 8 (Figure 10J) bare medially, with 14–15 medium-long to long hairs together with 1 to 2 slender short hairs on each side. Ovipositor valves (Figure 10J) triangular, thin, membranous, each moderately covered with micro-setae interspersed with 2 to 3 short hairs. Genital fork (Figure 10K) of usual inverted-Y form, with thick stem, arms of moderate width and moderately folded medially, and each lateral plate with thin projection directed posteromedially. Paraproct in ventral view (Figure 10L) with 3 or 4 sensilla on anteromedial surface; paraproct in lateral view (Figure 10M) slightly produced ventrally, 0.34–0.44 times as long as wide, with 23 or 24 medium-long to long hairs on ventral and lateral surfaces. Cercus in lateral view (Figure 10M) short, rounded posteriorly, 0.45–0.46 times as long as wide. Spermatheca (Figure 10N) ellipsoidal, 1.6 times as long as its greatest width.

**Figure 11:**
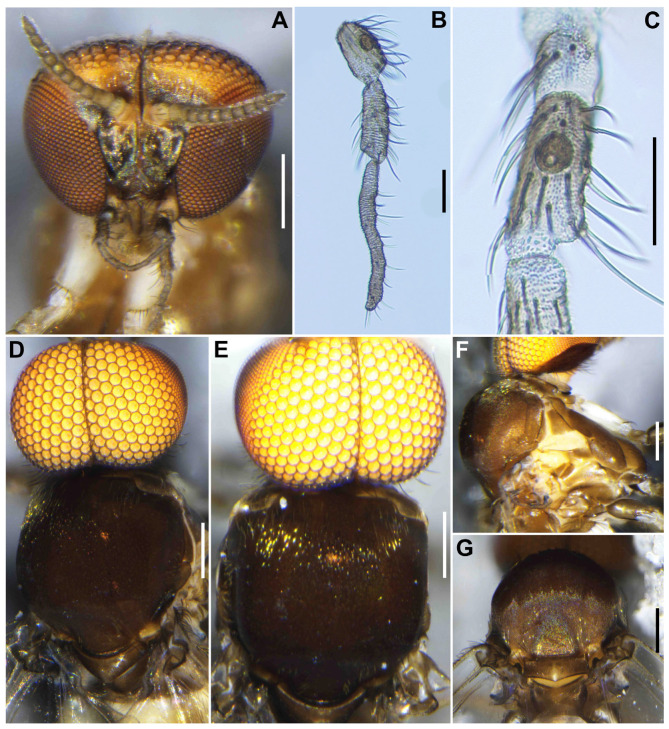
Male of *S. trangense*. (**A**) Head, front view; (**B**) Maxillary palpus, segments 3–5; (**C**) Third palpal segment with sensory vesicle; (**D**–**G**) Scuta, dorsal (**D**,**E**), lateral (**F**), and posterior (**G**) view, illuminated dorsomedially (**D**) and anteriorly (**E**). Scale bars: 0.2 mm for (**A**,**D**–**G**) and 0.05 mm for (**B**,**C**).

**Male** (*n* = 5) Similar to the male of *S*. *sipoense* sp. nov., except in following characters. Body length 2.3–2.6 mm (mean 2.5 mm). ***Head*** (Figure 11A). Upper eye bright medium brown, consisting of large facets in 11 vertical columns and 13 horizontal rows. Clypeus densely covered with whitish-yellow medium-long hairs (mostly directed upward) interspersed with several dark longer hairs on each side. Antenna brownish black except scape, pedicel, and base of first flagellomere yellow; first flagellomere elongate, 1.5 times length of second. Maxillary palpus light to medium brown, proportional lengths of third, fourth, and fifth segments 1.0:1.3–1.4:2.6–2.7 (Figure 11B); third segment (Figure 11C) widened apically; sensory vesicle (Figure 11C) ovoid, small, 0.28–0.29 times length of third segment, and with medium opening. ***Thorax*** (Figure 11D–G). Scutum brownish black, except shoulders yellow, scutum white pruinose on each shoulder, along lateral margins and on prescutellar area (Figure 11E–G), when illuminated at various angles. Katepisternum moderately covered with yellow and dark brown short hairs.

**Figure 12:**
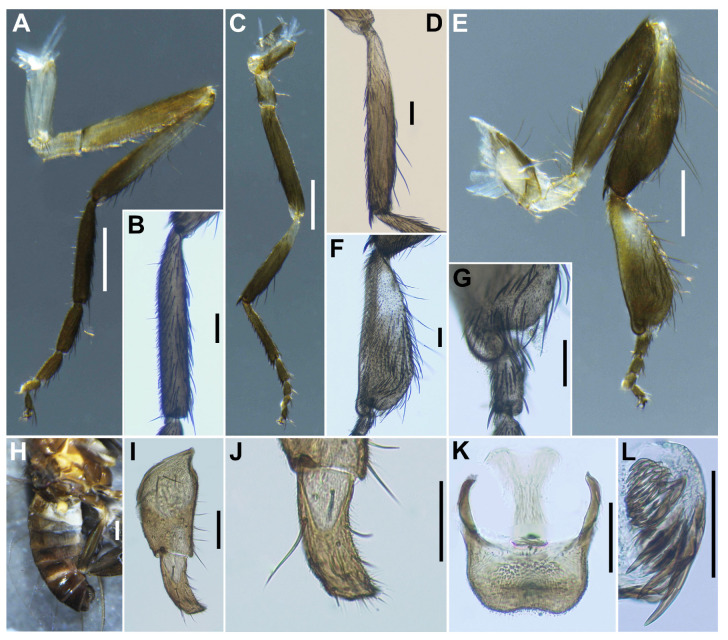
Male of *S. trangense*. (**A**) Foreleg; (**B**) Fore basitarsus; (**C**) Midleg; (**D**) Mid tibia; (**E**) Hind leg; (**F**) Hind basitarsus; (**G**) Calcipala, right side, inner view; (**H**) Abdomen, lateral view; (**I**) Coxite and style, ventral view; (**J**) Style, ventrolateral view; (**K**) Ventral plate and median sclerite, ventral view; (**L**) Paramere. Scale bars: 0.2 mm for (**A**,**C**,**E**,**H**) and 0.05 mm for (**B**,**D**,**F**,**G**,**I**–**L**).

***Legs***. Foreleg (Figure 12A), basitarsus (Figure 12B) nearly parallel-sided, 6.6 times as long as its greatest width. Midleg (Figure 12C), tibia (Figure 12D) whitish yellow on basal one-fifth, and medium to dark brown on rest; tarsus dark brown to brownish black, except basal two-fifths of basitarsus light to medium brown, though its border not well defined. Hind leg (Figure 12E), trochanter whitish yellow; femur light brown with basal extreme yellow and apical cap dark brown; tibia light to medium brown with base yellowish and apical cap brownish black; tarsus medium to dark brown except basal one-third of basitarsus white (though base medium brown) yellow (Figure 12F) and basal one-third of second tarsomere yellow; basitarsus (Figure 12F) enlarged, wedge-shaped, 2.87 times as long as wide, and 1.0 and 1.33 times as wide as greatest width of tibia and femur, respectively; calcipala (Figure 12G) shorter than basal width and 0.26 times as wide as greatest width of basitarsus. ***Wing*** (*n* = 10). Length 1.8–1.9 mm (mean 1.8 mm); other characteristics as in female, except subcosta without hairs. ***Halter***. White except basal stem darkened. ***Abdomen*** (Figure 12H). Basal scale black, with fringe of medium brown long hairs. ***Genitalia***. Coxite in ventral view (Figure 12I) nearly rectangular, 2.0 times as long as its greatest width. Style in ventrolateral view (Figure 12J) 1.8 times as long as its greatest width at the base, 0.63 times length of coxite, and nearly parallel-sided on basal one-third, gently narrowed toward apex. Ventral plate in ventral view (Figure 12K) with body transverse, 0.65 times as long as wide, parallel-sided and posterior margin depressed medially, densely covered with micro-setae on ventral surface; basal arms of moderate length, slightly divergent from base to middle, then somewhat convergent apically. Parameres (Figure 12L) each with 4 long and 1 medium-long hooks and several short ones. Aedeagal membrane moderately covered with micro-setae. Abdominal segment 10 without distinct hairs near posterolateral surface. Cercus encircled with 5–7 hairs.

**Figure 13:**
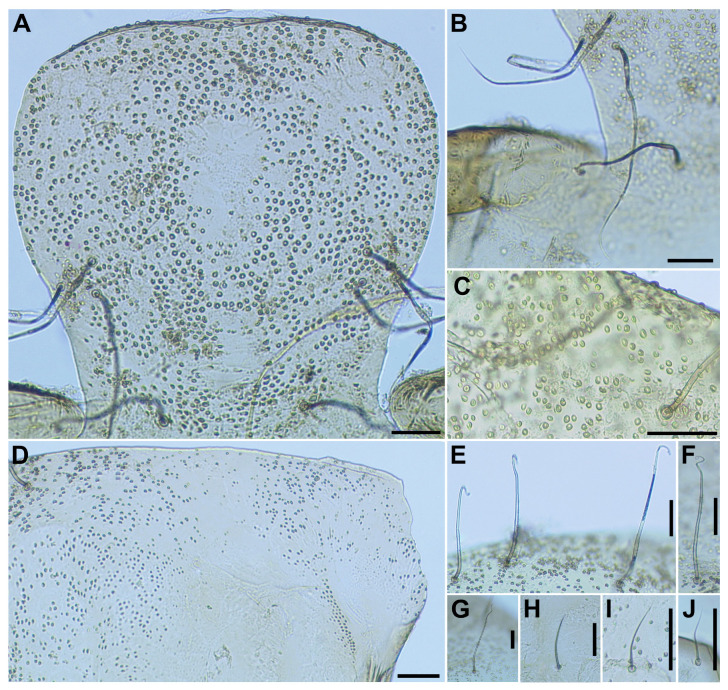
Pupa of *S. trangense*. (**A**) Frons and tubercles on frons; (**B**) Frontal trichomes and facial trichome; (**C**,**D**) Tubercles on thoracic integument of anterior (**C**) and posterior half (**D**) portions; (**E**–**J**) Thoracic trichomes on anterodorsal (**E**), anterolateral (**F**,**G**), mediolateral (**H**), and ventrolateral (**I**,**J**) surfaces. Two types of trichomes are present on anterolateral surface including coiled (**F**) and uncoiled (**G**) trichomes. Scale bars: 0.05 mm.

**Pupa** (*n* = 14). Body length 3.1–3.4 mm (mean 3.3 mm). ***Head***. (Figure 13A) Integument yellow, moderately covered with small round tubercles, except antennal sheaths. Frons with 3 pairs of unbranched long trichomes (Figure 13B); face with pair of unbranched long trichomes with coiled apex (Figure 13B); 3 frontal trichomes on each side arising close together, subequal in length to one another, and somewhat longer than facial one. ***Thorax***. Integument yellow, moderately covered by round tubercles on each anterior lateral portion (Figure 13C) and sparsely covered with small round tubercles on the posterodorsal portion (Figure 13D), with 3 long dorsomedial trichomes (Figure 13E), 2 anterolateral trichomes with coiled (Figure 13F) and uncoiled apices (Figure 13G), 1 medium-long mediolateral trichome with uncoiled apex (Figure 13H), and 3 ventrolateral trichomes with uncoiled apices (2 medium-long (Figure 13I), 1 short (Figure 13J)) on each side; all trichomes unbranched.

**Figure 14:**
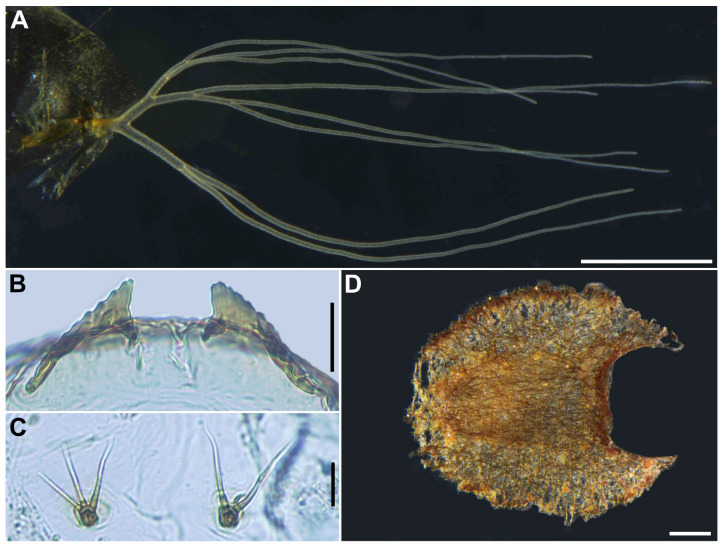
Pupa of *S. trangense*. (**A**) Gill filaments; (**B**) Terminal hooks, caudal view; (**C**) Bifid and trifid hooks on ventral surface of abdominal segment 5; (**D**) Cocoon, dorsal view. Scale bars: 0.5 mm for (**A**,**D**) and 0.02 mm for (**B**,**C**).

Gill (Figure 14A) composed of 8 slender thread-like filaments, arranged as [(2+1)+(1+2)]+2 from dorsal to ventral, with short common basal stalk having somewhat swollen transparent basal fenestra at base; common basal stalk 0.50 times length of interspiracular trunk; dorsal and middle triplets sharing medium-long stalk; dorsal triplet composed of 2 paired and 1 individual filaments with short primary and secondary stalks; middle triplet composed of 1 individual and 2 paired filaments with medium-long primary (as long as common stalk of dorsal and middle triplets) and short secondary stalks; ventral paired filaments with long stalk 1.7–2.1 times length of common basal stalk and as long as interspiracular trunk; all filaments light yellow, gradually tapered toward apex; 2 filaments of ventral pair subequal in length (ca. 2.7 mm including their own stalk and common basal stalk) to each other, 6 filaments of dorsal and middle triplets subequal in length to one another, and slightly shorter than 2 filaments of ventral pair; filaments of ventral pair subequal in thickness to each other; primary stalk of dorsal triplet lying against stalk of lower pair at angle of about 90 degrees when viewed laterally; cuticle of all filaments with well-defined annular ridges, densely covered with micro-tubercles. ***Abdomen***. Dorsally, all segments unpigmented or light yellow, though segments 5–8 somewhat lighter, and without micro-tubercles; first segment with 1 slender long hair-like seta on each side; segment 2 with 2 slender long hair-like setae and 5 short setae submedially on each side, all setae unbranched; segments 3 and 4 each with 4 hooked spines, 1 unbranched medium-long seta on segment 3 and 2 on segment 4 on each side; segment 9 with pair of wide, plate-like terminal hooks (Figure 14B), with their outer margins 2.6–2.7 times as long as their inner margins and crenulated. Ventrally, segment 4 with 1 unbranched hook; segment 5 with pair of bifid or trifid hooks (Figure 14C) submedially; segments 6 and 7 each with 1 medium-long seta and few unbranched slender short setae on each side. ***Cocoon*** (*n* = 4). Length 3.3–3.4 mm (mean 3.3 mm), width 2.4–2.8 mm (mean 2.6 mm) (Figure 14D).

**Figure 15:**
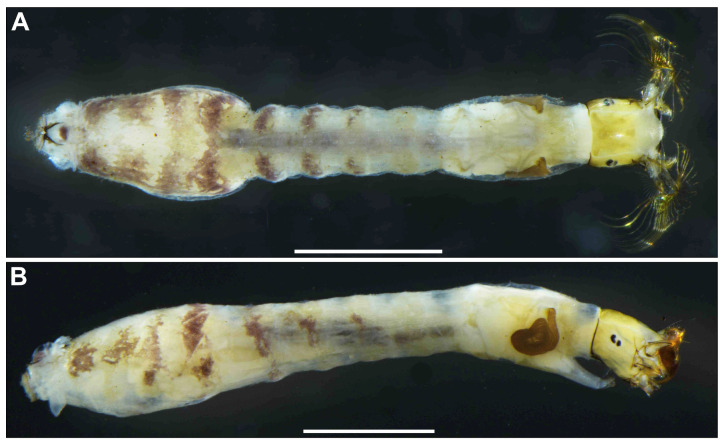
Larva of *S. trangense.* (**A**,**B**) Whole body, dorsal (**A**) and lateral (**B**) view. Scale bars: 1 mm.

**Mature larva** (*n* = 9). Body length 4.3–4.9 mm. (mean 4.6 mm) (Figure 15A,B). Yellowish, first thoracic segment with light gray transverse band unconnected ventrally. Abdominal segments 2–4 each with pair of reddish-brown dorsolateral markings, and abdominal segment 4 also with faint reddish-brown marking ventrally, segments 5–8 each with transverse reddish-brown band on dorsal and dorsolateral surfaces, although those bands on abdominal segments 6–8 often disconnected dorsomedially.

**Figure 16:**
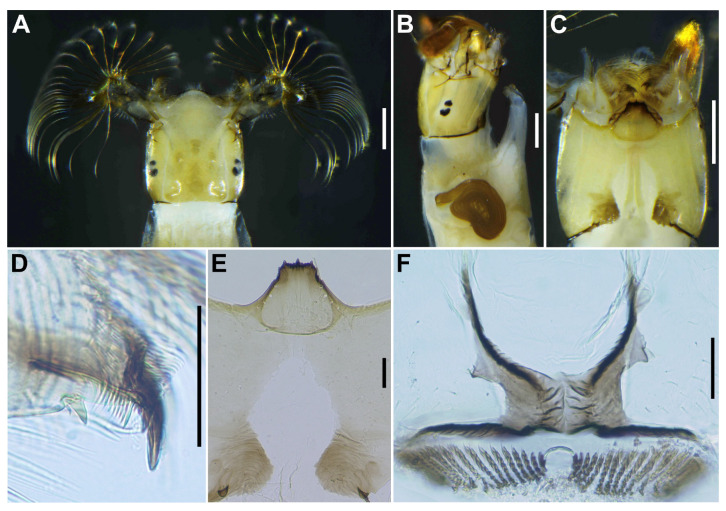
Larva of *S*. *trangense.* (**A**–**C**) Head capsules, dorsal (**A**), lateral (**B**), and ventral (**C**) view; (**D**) Mandible; (**E**) Postgenal cleft; (**F**) Anal sclerite, dorsal view. Scale bars: 0.2 mm for (**A**–**C**) and 0.05 mm for (**D**–**F**).

***Head***. Head capsule width 0.45–0.50 mm. (mean 0.47 mm), length 0.45–0.57 mm. (mean 0.52 mm). Cephalic apotome (Figure 16A) whitish yellow, sparsely covered with colorless fine setae, and with head spots faintly positive. Lateral surface of head capsule (Figure 16B) yellow except eye-spot region whitish, sparsely covered with colorless fine setae. Ventral surface of head capsule (Figure 16C) light yellow, except basal portions of both sides of postgenal cleft dark brown; sparsely covered with minute colorless setae. Antenna proportional lengths of first, second, and third articles 1.00:1.50–1.60:1.04–1.10. Labral fan with 34 primary rays. Mandible (Figure 16D) with 3 comb-teeth decreasing in length from first tooth to third; mandibular serration composed of 1 medium-size and 1 tiny teeth, major tooth at acute angle against mandible on apical side; supernumerary serration absent. Hypostoma with row of 9 apical teeth, of which median tooth slightly longer than each corner tooth; lateral margins smooth; 4 hypostomal bristles per side lying nearly parallel to lateral margin. Postgenal cleft (Figure 16C,E) deep, arrowhead-shaped, 6.1 times as long as the postgenal bridge. ***Thorax*** and ***Abdomen***. Nearly as in larva of *S*. *sipoense* sp. nov., except in following characters. Rectal organ compound, each of 3 lobes with 4–7 finger-like secondary lobules per lobe. Anal sclerite (Figure 16F) of usual X-form, with anterior arms 1.0–1.1 times as long as posterior ones. Posterior circlet with 69 or 70 rows of hooklets, with up to 8–10 hooklets per row.

#### 3.2.2. Specimens Examined

Two female, five males (with their associated pupal exuviae and cocoons) (preserved in 80% ethanol) reared from pupae, and nine mature larvae (all in 80% ethanol) collected from the type locality of *S. trangense* (a stream at Huai Yang waterfall, Thap Sakae district, Prachuap Khiri Khan province, western Thailand), 2-II-2024, by S. Thongsahuan.

#### 3.2.3. Biology

The pupae and larvae of *S. trangense* were collected from fallen leaves in a stream (width 30 cm, depth 10–15 cm, bed sandy, 21.5˚C, pH 7.3, partially shaded, elevation 69 m, N 11°37′33″ E 99°36′50″). The associated species were *S*. *angulistylum* Takaoka & Davies, 1995, *S*. *siamense* Takaoka & Suzuki, 1984 complex, *S*. *aureohirtum*, *S*. *tani* complex, and species in the *S*. *striatum* species-group.

#### 3.2.4. Remarks

This new species belongs to the *S. ceylonicum* species-group of the subgenus *Gomphostilbia* in the genus *Simulium*, redefined by Takaoka [1]. It is characterized by the dark hair tuft at the base of the radial vein and yellow fore coxae in both female and male, enlarged male hind basitarsi (Figure 12E,F) and ventral plate with its lateral margins nearly parallel-sided or slightly rounded (Figure 12K) when viewed ventrally, and the larval postgenal cleft, which is deep with its apex close to the posterior margin of the hypostoma (Figure 16C,E).

The female, male, pupa, and larva of this species are slightly different morphologically from those originally described (shown in parentheses) by Jitklang et al. [7] as follows: in the female, by the length ratio of the sensory vesicle against the third palpal segment 0.66 time (0.60 time); in the male, by the number of upper-eye facets, 11 vertical columns and 13 horizontal rows (9 or 10 vertical columns and 11 horizontal rows); in the pupa, by the primary stalk of the dorsal triplet against that of the ventral pair of filaments lying at an angle of 90 degrees (much wider than 90 degrees); and in the larva, by the postgenal cleft long, 6.1 time as long as postgenal bridge, but not reaching the posterior margin of the hypostoma (long, 34 times, almost reaching the posterior margin of the hypostoma).

*Simulium trangense* is closely similar to *S. sipoense* sp. nov. in sharing the following characteristics: in the female, large sensory vesicle; in the male, similar number of upper-eye large facets; in the pupa, similar shape of the terminal hooks; in the larva, abdominal segments 5–8 moderately covered with dark setae, each with 2–4 branches and long postgenal cleft deep. However, it is distinguished from *S. sipoense* sp. nov. (in parentheses) in the female by the hind tibia whitish yellow on the basal two-fifths or a little less than the basal half (whitish on the basal half or a little more), and by a shorter claw tooth 0.33 times as long as the claw (0.45); in the male, by the hind tibia dark except its base whitish (cf. dark except the basal one-third whitish), and by the ventral plate with its posterior margin depressed medially (produced medially); and in the larva, by the body yellowish (cf. gray) and thoracic segment 1 lacking a dark gray broad transverse band (cf. with a broad dark gray band).

*Simulium trangense* is distinguished from the four other Thai members (*S*. *curtatum*, *S*. *pangsidaense*, *S*. *sheilae*, and *S*. *wijiti*) of the *S. ceylonicum* species-group by certain characteristics: in the female, by a small basal tooth 0.33 times as long as the claw, shorter than all other species of the *S. ceylonicum* species-group (0.48–0.50 in *S*. *wijiti*; 0.50 in *S*. *sheilae*; 0.53 in *S*. *pangsidaense*; and data unavailable for *S*. *curtatum*); in the male, by the upper-eye (large) facets arranged in 11 vertical columns and 13 horizontal rows, differing from other species (13 columns and 14–16 rows in *S*. *curtatum* and *S*. *pangsidaense*; 10 columns and 13 rows in *S*. *sheilae*; and 15 columns and 15 or 16 rows in *S*. *wijiti*), length ratio of the hind basitarsus against its greatest width 2.9 time, compared to 3.5–4.6 time in *S*. *pangsidaense, S*. *sheilae*, and *S*. *wijiti* (data unavailable for *S*. *curtatum*); in the pupa, by the terminal hook, which is wide and plate-like with its outer margin crenulated, except in *S. pangsidaense* (terminal hook triangular, with its outer margin smooth or slightly undulate in *S. sheilae* and *S. wijiti*; data unavailable for *S*. *curtatum*); in the larva, by the number of the primary rays of the labral fan 34, compared to 47 in *S*. *sheilae* and 27–32 in *S*. *wijiti* and *S*. *pangsidaense* (data unavailable for *S*. *curtatum*). The first abdominal segment lacks a reddish-brown transverse band, in contrast to *S. sheilae* and *S. pangsidaense* (which have a reddish-brown band) and *S. curtatum* and *S. wijiti* (which have a greenish transverse band) [5,7,21,22].

*Simulium trangense* is distinguished from *S. doisaketense* and *S.* nr. *sheilea* sp. 3, both reported from northern Thailand by Jitklang et al. [7], in the larval stage by the presence of reddish-brown transverse bands on abdominal segments 2–4 and a deep postgenal cleft that is 5.5 times as long as the postgenal bridge. In contrast, *S. doisaketense* and *S.* nr. *sheilea* sp. 3 have greenish transverse bands on abdominal segments 1–4 and a medium-long postgenal cleft (1.5–2.3 times as long as the postgenal bridge).

*Simulium trangense* is similar to *Simulium* sp. nr. *asakoae* 4, which was reported from Thailand by Jitklang et al. [7], in having a long postgenal cleft, with its apex almost reaching the posterior margin of the hypostoma in the larva, and a crenulated outer margin of the terminal hook in the pupa. However, it is distinguished from *S.* sp. nr. *asakoae* 4 by the presence of reddish-brown markings on the dorsolateral surface of the larval abdomen, which are absent in the latter species.

*Simulium trangense* is similar to *S. leparense* from Malaysia in many characteristics, such as the number of inner and outer teeth on the mandible in the female, the number of parameral hooks in the male, and a short common stalk of the gill with 8 filaments and terminal hooks that are wide, plate-like, with crenulated outer margins in the pupa. However, *S. trangense* can be differentiated from *S. leparense* by the following characteristics (characteristics of *S. leparense* in parentheses): in the female and male, by the color of short hairs on the scutum, which are whitish yellow (dark brown); in the female, by the length ratio of the sensory vesicle to the third palpal segment of 0.66 (0.55) and shorter basal tooth 0.33 times as long as claw (0.5 time); in the male, by the number of upper-eye (large) facets arranged in 11 vertical columns (7 or 8); and in the pupa, by the presence of 3 grapnel-shaped hooklets on each side of abdominal segment 9 (absent) [23].

*Simulium trangense* appears to be similar in the female to *S. namense* Takaoka, 1989, which was described from Myanmar by Takaoka [24], in having a brownish-black to black scutum (with anterolateral whitish-pruinose calli). However, it differs from *S. namense* (characteristics of *S. namense* in parentheses) in the female by the number of mandibular teeth, with 18 inner and 9 or 10 outer teeth (26 inner and 3 or 4 outer teeth), elongate sensory vesicle that is 0.66 times as long as the third segment (small, ovoid, and about 0.25 times as long as the third segment), and a small basal tooth 0.33 times as long as the claw (0.5 time); in the male, by the number of upper-eye (large) facets arranged in 11 vertical columns and 13 horizontal rows (14 vertical columns and 15 horizontal rows) [24].

### 3.3. Genetic Analysis

A total of 31 *COI* sequences of the *S. ceylonicum* species-group were used for genetic analysis, of which two sequences of *S. sipoense* sp. nov. and three sequences of *S. trangense* were generated in this study. Sequence similarity searching with BLAST revealed that the new species is genetically closest to the species redescribed as *S. trangense* from Malaysia, with the highest similarity (99.54–99.85%) to the accession no. KM410182. The generated *COI* sequences of *S. trangense* redescribed in this study showed 98.98–100% similarity with the sequences of *S. sheilae* from Thailand (accession nos. HM775272–HM775274) and matched perfectly with the accession no. HM775272.

**Figure 17:**
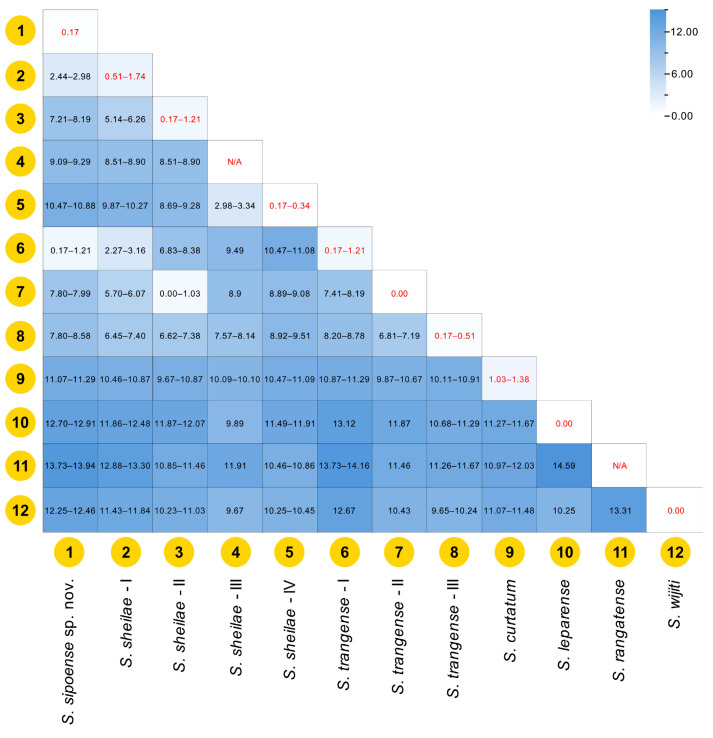
Color heatmap of K2P pairwise genetic distances (%) among species in the *S. ceylonicum* species-group. The color shading illustrates the maximum genetic distance for each pairwise comparison. Genetic distances are presented as ranges, with intraspecific values shown in red and interspecific values in black.

The K2P genetic distances among species of the *S. ceylonicum* species-group were summarized in Figure 17. Overall, the intraspecific genetic distances varied between 0% and 1.74%, with the greatest intraspecific genetic distance in *S. sheilae* lineage I. The interspecific genetic distances ranged from 0.17% (the new species versus *S. trangense* lineage I) to 14.59% (*S. leparense* versus *S. rangatense*). The intra- and interspecific genetic distances of the new species were 0.17% and 0.17–13.94%, respectively. Meanwhile, the intra- and interspecific genetic distances of *S. trangense* (lineage II) redescribed in this study were 0.00% and 0.00–11.87%, respectively.

**Figure 18:**
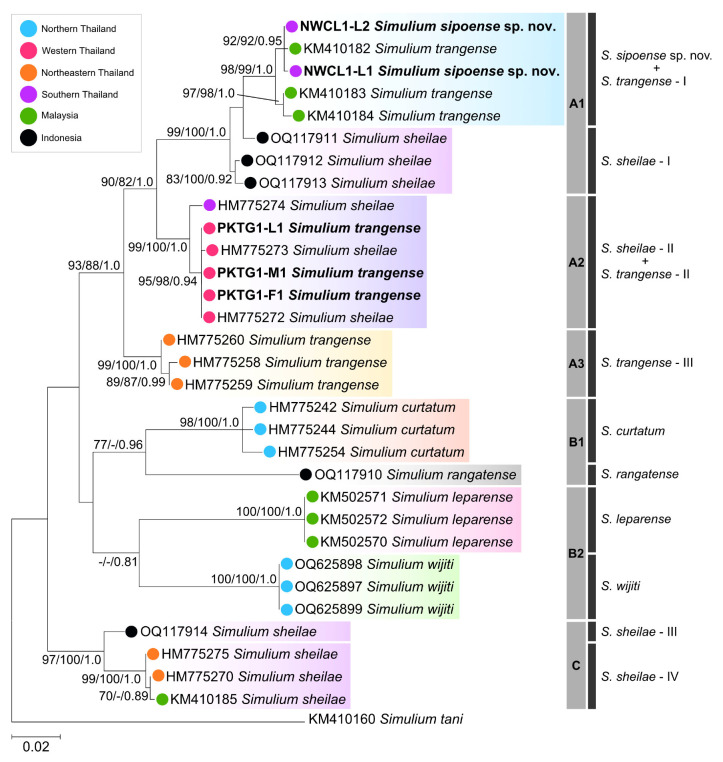
Maximum likelihood phylogenetic tree of the *S. ceylonicum* species-group based on 586 bp of the *COI* gene, with *S. tani* as an outgroup. The numbers near each branch correspond to bootstrap and posterior probability values (ML/NJ/BI), and only support values of ≥70% for ML and NJ or ≥0.70 for BI are indicated. New sequences generated in this study are in bold. Different colored circles before each sequence indicate the countries where black fly samples were collected. The light gray vertical bars at the tips of the tree represent the clades and subclades defined in this study, while the dark gray vertical bars indicate the recognized morphospecies and putative cryptic species reported from previous studies [3,4,25] and this study.

The phylogenetic analysis inferred from the *COI* gene produced consistent tree topologies across all methods (NJ, ML, and BI), placing all seven species of the *S. ceylonicum* species-group into three main clades (A, B, and C) (Figure 18), each comprising different species and/or lineages. Within these, *S. curtatum*, *S. rangatense*, *S. leparense*, and *S. wijiti* were recovered as monophyletic, while *S. trangense*, *S. sheilae*, and *S. sipoense* sp. nov. were non-monophyletic. Notably, *S. sheilae* and *S. trangense* were further divided into four and three lineages, respectively, some of which corresponded to their geographical distributions.

Clade A, a highly supported clade, comprised three species: *S. trangense* (lineages I-III), *S. sheilae* (lineages I and II), and *S. sipoense* sp. nov. This clade was separated into three subclades (A1, A2, and A3). Subclade A1 included *S. sheilae* lineage I from Indonesia, *S. trangense* lineage I from Malaysia, and *S. sipoense* sp. nov. from Thailand. In this subclade, the new species formed a well-supported group with *S. trangense* lineage I, closely related to *S. sheilae* lineage I. Subclade A2 was composed of *S. trangense* lineage II redescribed in this study and *S. sheilae* lineage II from southern and western Thailand, both clustered together with strong supports, showing a sister relationship to subclade A1. Subclade A3, the most basal subclade within clade A, was formed by a single species, *S. trangense* lineage III from northeastern Thailand.

Clade B, with low support, consisted of four species (*S. curtatum*, *S. leparense*, *S. rangatense*, and *S. wijiti*), and was further divided into two subclades (B1 and B2). Subclade B1 included a well-supported group of *S. curtatum* and a single sequence of *S. rangatense*, forming a sister relationship to subclade B2, which was composed of *S. leparense* and *S. wijiti*, both of which were fully supported groups.

Clade C, a well-supported and most basal clade, comprised only one species, *S. sheilae*, which was further divided into two lineages (III and IV), and was sister to the two larger clades (A and B).

## 4. Discussion

Originally, *S. trangense* was briefly described by Jitklang et al. [7] and later fully redescribed by Takaoka et al. [6], based on specimens collected from Langkawi Island, Malaysia. However, DNA barcoding studies suggested that *S. trangense* from Malaysia are genetically different from that of Thailand [3,4], raising the question of whether the two populations are conspecific or represent different species. In this study, using specimens of *S. trangense* collected from its type locality, we clearly confirmed that the two populations are indeed different species, which are both morphologically and genetically distinct. Morphologically, *S. trangense* is almost indistinguishable from several members of the *S. ceylonicum* species-group, but detailed morphological examinations revealed unique morphological traits that can be used to distinguish it from closely related species. These differences are evident in the female by a relatively shorter claw tooth; in the male, by the ventral plate being parallel-sided and its posterior margin depressed medially; in the pupa, by the terminal hook; and in the larva, by the abdominal banding patterns. Blast search and phylogenetic analysis revealed that *S. trangense* redescribed in this study is conspecific with *S. sheilae* (accession nos. HM775272–HM775274), collected from several localities in Thailand, including the type locality in Prachuap Khiri Khan [25]. *COI* DNA barcoding is very effective in differentiating species and revealing cryptic biodiversity within the *S. ceylonicum* species-group [3,4]. Thus, we strongly believe that the specimens previously identified as *S. sheilae* (lineage II) are actually true *S. trangense* (lineage II) that were morphologically misidentified. Furthermore, the previous study used larval specimens for molecular analysis, which are morphologically difficult to differentiate from other related species in the *S. ceylonicum* species-group, especially *S. trangense*.

Following the redescription of *S. trangense* in this study, the specimens from Malaysia previously used to redescribe this species in Thailand need to be re-examined both morphologically and molecularly. Although no specimens of the so-called *S. trangense* from Malaysia were available for the current re-analysis, our morphological and molecular examinations of newly collected specimens from Narathiwat province, Thailand, strongly supported that they are conspecific. Based on these results, the specimens from Narathiwat were used for the description of a new species, *S. sipoense* sp. nov., in this study. The new species is morphologically similar to several members of the *S. ceylonicum* species-group and is genetically closest to *S. sheilae* (lineage I) from Indonesia. Whether *S. sheilae* lineage I is the same new species or a new cryptic species requires further integrative taxonomic study.

The results of this study demonstrate that DNA barcoding is a highly effective tool for recognizing cryptic species within the *S. ceylonicum* species-group, leading to the description of *S. sipoense* sp. nov. and the redescription of *S. trangense*. Therefore, morphologically defined *S. trangense* and *S. sheilae* specimens that are genetically different from those collected at their type localities should be re-examined using an integrative morphological and molecular approach.

## 5. Conclusions

The discovery of a new species within the morphologically recognized *S. trangense*, along with the redescription of *S. trangense*, represents an interesting case study in black fly taxonomy. These findings suggest that both the redescription of known species and the description of new species should be carried out with caution, using integrated morphological and molecular taxonomic approach. Based on molecular evidence, several cryptic species currently identified morphologically as *S. sheilae* and *S. trangense* await clarification of their species status as to whether they are conspecific or represent biological distinct species.

## Figures and Tables

**Table 1:** Summary of *COI* DNA sequences for seven species of the *S. ceylonicum* species-group included in the molecular analysis.

Species	No.	Collection Site	Country	Accession No.	Reference
*S. sipoense* sp. nov.	2	Ra-ngae, Narathiwat	Thailand	PV938055	This study
				PV938056	
*S. curtatum*	3	Chom Thong, Chiangmai	Thailand	HM775242	Pramual et al. [3]
				HM775244	
		Khun Yuam, Mae Hong Son		HM775254	
*S. leparense*	3	Jerantut, Pahang	Malaysia	KM502570	Low et al. [20]
				KM502571	
				KM502572	
*S. rangatense*	1	Rangat, Kempo, Flores	Indonesia	OQ117910	Hew et al. [4]
*S. sheilae*	10	Phu Sing, Sisaket	Thailand	HM775270	Pramual et al. [3]
		Thap Sakae, Prachuap Khiri Khan		HM775272	
				HM775273	
		Mueang, Ranong		HM775274	
		Phu Pha Kham, Mukdahan		HM775275	
		Bentong, Pahang	Malaysia	KM410185	Low et al. [20]
		Lembah Harau, West Sumatra	Indonesia	OQ117911	Hew et al. [4]
				OQ117912	
				OQ117913	
				OQ117914	
*S. trangense*	9	Thap Sakae, Prachuap Khiri Khan	Thailand	PV177263	This study
				PV177264	
				PV177265	
		Na Khu, Kalasin		HM775258	Pramual et al. [3]
		Thep Sathit, Chaiyaphum		HM775259	
		Phu Pha Kham, Mukdahan		HM775260	
		Cameron Highlands, Pahang	Malaysia	KM410182	Low et al. [20]
				KM410183	
				KM410184	
*S. wijiti*	3	Pai, Mae Hong Son	Thailand	OQ625897	Srisuka et al. [5]
				OQ625898	
				OQ625899	

## Data Availability

All data supporting reported results are included in the text. The newly generated *COI* sequences were submitted to the GenBank database under the accession numbers PV177263–PV177265 for *S. trangense* and PV938055 and PV938056 for *S. sipoense* sp. nov.
